# Scopolamin-Morphin Anesthesia*Notes on Anesthetics, with Special Reference to Scopolamin-Morphin Anesthesia. A. C. Wood, Philadelphia, in *American Medicine*, Philadelphia, November 11th.

**Published:** 1905-12

**Authors:** 


### Scopolamin-Morphin Anesthesia.*

Wood epitomizes his personal experience with scopolamin-morphin anesthesia as follows: It is capable, in many cases, of producing a satisfactory surgical narcosis which lasts several hours. When successful, the patient avoids the anxiety or even alarm often felt before taking ether, and the nausea, vomiting, and depression following its administration. If it should partly or wholly fail to anesthetize the patient, the surgeon may use ether or chloroform, with the assurance that the effect will be more prompt and satisfactory than when either is administered alone. When used in conjunction with ether or chloroform, it has been especially satisfactory. The time required to induce anesthesia was lessened, relaxation promoted, the secretion of mucus in the respiratory tract prevented, and, what is very important, the quantity of anesthetic required was greatly reduced. To induce full anesthesia in the average healthy adult, Wood recommends 0.0006 gm. (1/100 gr.) scopolamin and 0.01 gm. (1/6 gr.) morphin, to be given hypodermically two or two and a half hours before operation, and a second similar dose an hour later. If the anesthesia is not sufficiently complete half an hour before operation, a third dose may be given. This may be the same as the two previous doses, or may consist of either drug alone, or of scopolamin in conjunction with morphin, according to the special indications present. The doses should be considerably reduced in children, feeble patients, and in advanced age. None of Wood's patients remembered feeling any pain, or any other detail of the operation, although in some instances the patient gave evidences of the presence of sensation at the time. This preparation is contra-indicated in acute affections of the pharynx and larynx; in operations involving the mouth or air passage; in edema of the lungs, and in cases in which capillary hemorrhage may be a troublesome factor. As both scopolamin and morphin are powerful drugs, prudence demands that they be used with caution. In addition to the caution mentioned, the safety and success of the method depend on having pure and reliable drugs, accurate dosage, and perfectly fresh solutions.—*The Octopus.*

*Notes on Anesthetics, with Special Reference to Scopolamin-Morphin Anesthesia. A. C. Wood, Philadelphia, in *American Medicine,* Philadelphia, November 11th.

